# An unusual presentation revealing Peutz-Jeghers syndrome in adult

**DOI:** 10.1016/j.amsu.2020.08.034

**Published:** 2020-09-01

**Authors:** Seifeddine Ben Hammouda, Manel Njima, Nouha Ben Abdeljelil, Ahlem Bellalah, Leila Njim, Abdelfattah Zakhama

**Affiliations:** a Department of Pathology, Fattouma Bourguiba University Hospital, Monastir, 5000, Tunisia; b Faculty of Medicine, University of Monastir, Monastir, 5000, Tunisia

**Keywords:** Intussusception, Hamartomatous polyp, Peutz-Jeghers syndrome, Intestinal obstruction

## Abstract

**Introduction:**

Peutz-Jeghers syndrome (PJS) is a rare hereditary disease characterized by hyperpigmentation on the lips and oral cavity and gastrointestinal hamartomatous polyps. The most common complications in PJS patients are bleeding, bowel obstruction and intussusception.

**Presentation of case:**

We hereby report a case of a 33-year-old female, without a family history of the disease, who presented to the emergency room with acute abdominal pain, bloating and not passing gas. On abdominal examination, upper abdominal and periumbilical tenderness was found. Computed tomography (CT) of the abdomen demonstrated suspected ascending colon intussusception. The patient underwent a mid-line laparotomy that showed an ileocolic intussusception. Reduction of this intussusception was successfully done with resection of the affected segment that showed presence of two pedunculated polyps. The specimen was sent thereafter to our department for histopathological evaluation, which confirmed the diagnosis of hamartomatous Peutz-Jeghers polyps with no malignancy. Afterwards, the patient was carefully reexamined and the physical examination revealed multiple pigmented spots on the face and lips. Thus, the diagnosis of Peutz-Jeghers syndrome was made.

**Discussion:**

PJS is a rare autosomal dominant disorder that often remain undiagnosed for many years. Acute complications such as intestinal obstruction secondary to intussusception is one of infrequent revealing symptoms.

**Conclusion:**

Early identification, in patients with PJS and family members, as well as close cancer surveillance can improve certainly prognosis in these individuals.

## Highlights

• Peutz-Jeghers syndrome is a rare genetic disorder that often remain undiagnosed for many years.
• Complications of this disease, such as intussusception, are rarely seen thanks to the facility of digestive endoscopic polypectomy.
• Intestinal obstruction secondary to intussusception is one of infrequent revealing symptoms.
• Early identification, in patients with PJS and family members, as well as close cancer surveillance can improve certainly prognosis in these individuals.

## 1 Introduction

Peutz-Jeghers syndrome (PJS) is a rare inherited autosomal dominant disease, with an estimated prevalence from 1 in 100,000 people [1]. It was first described by Peutz in 1921 and Jeghers in 1944 and 1949 [2]. This disorder is seen equally in both male and female patients and usually diagnosed during childhood or early adulthood [3,4]. It is characterized by the development of gastrointestinal hamartomatous polyps and mucocutaneous pigmentations [5]. Bleeding, bowel obstruction and intussusception are common complications in PJS patients [6]. Although the hamartomatous polyps are known with low malignant potential, individuals with this disease are at elevated risk of acquiring malignancies [7]. Here, we present an interesting case of a young woman with Peutz-Jeghers syndrome.

This work has been reported in line with the SCARE criteria [8].

## 2 Presentation of a case

**Fig. 1:**
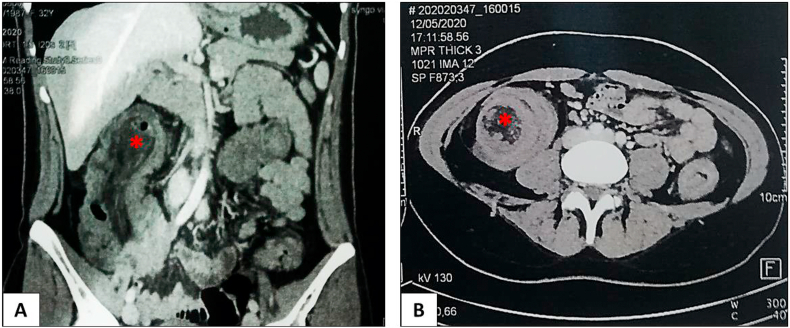
(A): CT of abdomen showed ileocolic intussusception (asterisks), which extended over 18cm with “target sign” (B).

**Fig. 2:**
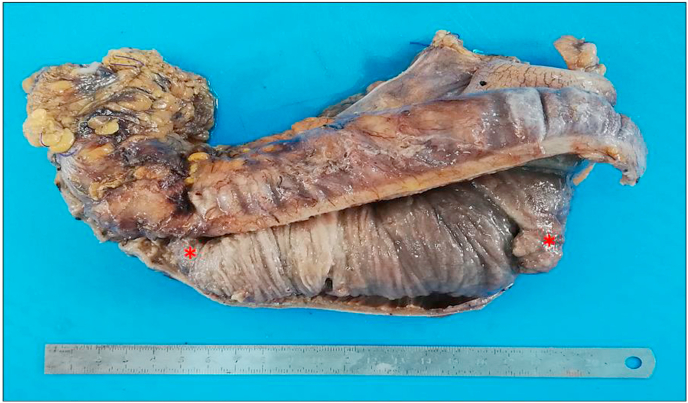
Gross examination of the ileocolic resection specimen. At the opening, it showed a segment of small intestine invaginating into the adjoining intestinal lumen consistent with an intussusception that had caused large bowel obstruction. This segment had two pedunculated polyps measuring 3 × 1.5cm and 3.5 × 2cm (asterisks).

A 33-year-old female, with no significant medical history nor family history of genetic disease, visited the Emergency Department for acute abdominal pain, bloating and not passing gas. Her vital signs were normal. On abdominal examination, upper abdominal and periumbilical tenderness was found. Computed tomography (CT) of the abdomen was performed and demonstrated suspected ascending colon intussusception (Fig. 1). The patient underwent a mid-line laparotomy, by a university assistant in general surgery, which showed an ileocolic intussusception. Reduction of this intussusception was successfully done with resection of the affected segment that showed presence of two pedunculated polyps. The postoperative course was uneventful. The specimen was sent thereafter to our department for histopathological evaluation. On gross examination, we received an ileocolic resection specimen, which was comprised of colonic segment measuring 25 cm in length, an ileal segment measuring 4 cm in length and an appendix that measured 8 cm in length and 0.5 cm in diameter. On opening the specimen, we found a segment of small intestine invaginating into the adjoining intestinal lumen with blackish appearance and measuring 19 cm in length and 4 cm in diameter consistent with an intussusception that had caused large bowel obstruction. This segment had two pedunculated polyps measuring 3 × 1.5cm and 3.5 × 2cm and located respectively 6 cm and 18cm from the ileal margin. The resection margins were free with no malignancy (Fig. 2).

**Fig. 3:**
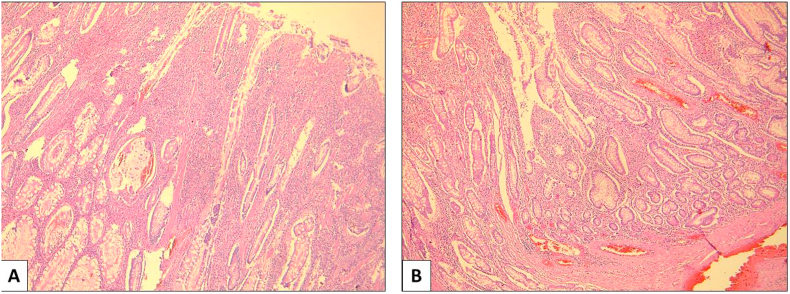
Histological findings, hematoxylin and eosin (x100). A, B: Peutz-Jeghers polyp characterized by villous architecture and arborizing smooth muscle cores. The mucosa covering the polyp was hyperplastic and often ulcerated, with no sign of malignancy.

Microscopically, these polyps corresponded to benign hamartomatous polyps that showed branching muscular core formed of smooth muscles with covering hyperplastic and ulcerated mucosa (Fig. 3). Based on the above features, the diagnosis of intestinal intussusception caused by small bowel hamartomatous Peutz-Jeghers polyps with no malignancies was given. Afterwards, the patient was carefully reexamined and the physical examination revealed multiple pigmented spots on the face and lips. Thus, the diagnosis of Peutz-Jeghers syndrome was made. Regular assessment of our patient is maintained as recommended and she is adhering for her screening program.

## 3 Discussion

PJS is an autosomal dominant disease, which is characterized by the development of hamartomatous polyps in the gastrointestinal tract and mucocutaneous lentiginosis [9]. This lesion is seen mostly on the lips, oral, and gingival mucosae [10]. The incidence of PJS is reported to be 1 in 100,000 individuals [1]. This disorder is seen equally in both male and female patients and usually diagnosed during childhood or early adulthood [3,4]. Inheritance of this syndrome is autosomal dominant with incomplete penetrance. Mutations to the serine/threonine kinase 11 (STK11) tumor suppressor gene on chromosome 19p13.3 have been shown to cause Peutz-Jeghers syndrome [11].

The morbidity of this syndrome is due primarily to lesions of the small intestine [12] that generally require iterative enterectomies [13]. Actually, complications of this procedure, such as intussusception, are rarely seen thanks to the facility of digestive endoscopic polypectomy [14]. Clinically, PJS patients often present with a history of intermittent abdominal pain, which is due to bowel intussusception caused by the polyps. This may be reduced spontaneously or develop acute bowel obstruction. Individuals affected with PJS may present also with acute blood loss and chronic anemia due to the ulceration of polyps [15]. The diagnosis of PJS should be made, according to the World Health Organization (WHO), in a patient who presents any one of the following criteria: “i) Three or more Peutz-Jeghers polyps confirmed by histology; ii) Any number of Peutz-Jeghers polyps with a family history of the syndrome; iii) Typical visible mucocutaneous pigmentation with a family history of the syndrome; iv) Any number of Peutz-Jeghers polyps and typical visible mucocutaneous pigmentation” [16].

Lentiginosis of the lips and oral mucosa is an essential feature for early diagnosis.

It usually appears in childhood but in adulthood may fade and even disappear [17]. Our patient had the classic PJS characterization of oral hyperpigmentation and the diagnosis of hamartomatous Peutz-Jeghers polyps was made by the histopathological examination. Therefore, the diagnosis was retained according to the fourth WHO′ s criteria. For pathological features, the macroscopic appearance of Peutz-Jeghers polyps is not distinctive. Histologically, they are characterized by villous architecture and arborizing smooth muscle cores. Prolapse and peristaltic kneading is relatively common in Peutz-Jeghers polyps causing epithelial misplacement and may extend into the serosa, mimicking a well-differentiated invasive lesion [18].

Small bowel intussusception can be a major source of mortality for patients with PJS. However, it can be prevented with push enteroscopy and polypectomy to avoid multiple surgical resections, which lead to short bowel syndrome [18].

Another aspect in Peutz-Jeghers patients is the high risk of occurrence or progression of intestinal and extraintestinal malignant tumors. The frequency of this risk in these patients is higher than in the general population [19]. Nevertheless, there is no proof until now whether the neoplastic lesions are due to transformation of hamartomatous polyps [20]. The reported lifetime risk for any cancer varied according to the literature between 37 and 93% at the age of 60–70 years [21]. The most common malignant neoplasm associated with PJS reported are: gastrointestinal, gynecological, pancreatic, and lung [21]. Thus, early identification and careful screening of patients with PJS is recommended to prevent gastrointestinal complications and cancers. Specific testing should include monitoring of hemoglobin levels, as well as regular gynecologic, pelvic and testicular examinations and gastrointestinal tract screening with upper and lower endoscopy in combination with small bowel video capsule endoscopy [22].

## 4 Conclusion

PJS is a rare autosomal dominant disorder that often remain undiagnosed for many years. Acute complications such as intestinal obstruction secondary to intussusception is one of infrequent revealing symptoms. Thus, early identification, in patients with PJS and family members, as well as close cancer surveillance can improve certainly prognosis in these individuals.

## Sources of funding

None.

## Ethical approval

Exemption from ethnical approval.

## Consent

The patient accepted the publication of his case report.

## Author contribution

Seifeddine Ben Hammouda: data analysis and writing the paper.

Manel Njima and Nouha Ben Abdeljelil: bibliography, coordination and helped to draft the manuscript.

Ahlem Bellalah: specimen contribution and data collection.

Leila Njim and Abdelfatteh Zakhama: revision.

## Registration of research studies

This is not applicable to our case report.

## Guarantor

Seifeddine Ben Hammouda.

## Provenance and peer review

Not commissioned, externally peer reviewed.

## Declaration of competing interest

None.
